# A New Method of Pivoting

**Published:** 1874-02

**Authors:** B. Bannister


### A NEW METHOD OF PIVOTING.

BY B. BANNISTER.

I have recently reset several pivot teeth (having previously worn the wood pivot for a number of years) upon a method I think new and quite practical, and will attempt a description.

My first experiment was upon a superior lateral incisor. The root, after all decay and softened portions were removed, represented a large funnel shaped opening, nearly to the end, for the reception of pivot, with a destruction of end of root for crown attachment under the gum, fully two lines. I selected a plate tooth for form, color, etc., as desired, and fitted to anterior portion of root, not being particular, as the final part of the operation will remedy any defect here. I then rivet a gold backing to the tooth, using gold as thick as the rivets will admit. Let the backing extend in direction of pivot, nearly the length I desire it when finished. The next work to be done is to obtain a good impression of the root. Soften gutta percha and fill the root a trifle less than full, or try to approximate the amount of displacement of gutta percha caused by pivot when inserted. (A surplus is required to restore any lost part, and as a base for tooth. Any more than required is detrimental.) Heat the gold pivot or backing, press through the gutta percha to the position you wish the tooth to have when completed. When cool, remove carefully. Carve with wax any desired shape, to give form to tooth or accomodate the "bite." Make a mould in plaster, remove the wax and gutta percha, fill with rubber and vulcanize. Polish such parts as require no fitting or adjusting. If the work has been done correctly, in having a true impression and closing flask, it will be a success.

I have inserted several under very unfavorable conditions for pivoting by other methods, with good results. The manipulations may be improved and should be. Something better than gutta percha is desirable for impressions. Two cases I had to revulcanize, which I attributed to giving a little lateral motion in holding the tooth waiting for it to cool (still it may have been from other causes). In each trial I see some little improvement, and, while giving my method, I only wish to furnish the idea of a rubber base and pivot to be improved upon. I think it has value. If the method is practicable, it will readily be seen where it is needed. Give it a trial.

